# A phase II JN-I-10 efficacy study of IDRF-based surgical decisions and stepwise treatment intensification for patients with intermediate-risk neuroblastoma: a study protocol

**DOI:** 10.1186/s12887-020-02061-5

**Published:** 2020-05-12

**Authors:** Tomoko Iehara, Akihiro Yoneda, Atsushi Kikuta, Toshihiro Muraji, Kazuaki Tokiwa, Hideto Takahashi, Satoshi Teramukai, Tetsuya Takimoto, Shigeki Yagyu, Hajime Hosoi, Tatsuro Tajiri, the Japan Children’s Cancer Group Neuroblastoma Committee

**Affiliations:** 1 Department of Pediatrics, Graduate School of Medical Science, Kyoto Prefectural University of Medicine, Kajii-cho, Kawaramachi-Hirokoji, Kamigyo-ku, Kyoto, 602-8566 Japan; 2 Department of Pediatric Surgery, Osaka City General Hospital, Osaka, Japan; 3 Department of Pediatric Oncology, Fukushima Medical University Hospital, Fukushima, Japan; 4 Department of Pediatric Surgery, Kirishima Medical Center, Kagoshima, Japan; 5 Department of Pediatric Surgery, Kyoto Prefectural University of Medicine, Graduate School of Medical Science, Kyoto, Japan; 6 National Institute of Public Health, Saitama, Japan; 7 Department of Biostatistics, Kyoto Prefectural University of Medicine, Graduate School of Medical Science, Kyoto, Japan; 8 Clinical Epidemiology Research Center for Pediatric Cancer, National Center for Child Health and Development, Tokyo, Japan

**Keywords:** Neuroblastoma, Intermediate risk, IDRF

## Abstract

**Background:**

Few clinical trials have been reported for patients with intermediate-risk neuroblastoma because of the scarcity of the disease and the variety of clinical and biological characteristics. A multidisciplinary treatment that consists of multidrug chemotherapy and surgery is expected to lead to a good prognosis with few complications. Therefore, a clinical trial for patients with intermediate-risk tumors was designed to establish a standard treatment that reduces complications and achieves good outcomes.

**Methods:**

We planned a prospective phase 2, single-arm study of the efficacy of image-defined risk factors (IDRF)-based surgical decision and stepwise treatment intensification for patients with intermediate-risk neuroblastomas. For the localized tumor group, IDRF evaluations will be performed after each three-course chemotherapy, and surgery will be performed when appropriate. For patients with metastatic tumors, a total of five chemotherapy courses will be performed, and primary lesions will be removed when the IDRF becomes negative. The primary endpoint is 3-year progression-free survival rate, and the secondary endpoints include 3-year progression-free survival rates and overall survival rates of the localized group and the metastasis group and the incidence of adverse events. From international results, 75% is considered an appropriate 3-year progression-free survival rate. If this trial’s expected 3-year progression-free survival rate of 85% is statistically greater than 75% in the lower limit of the 95.3% confidence interval, with an accuracy 10% (85 ± 10%), both groups require more than 65 patients.

**Discussion:**

This study is the first clinical trial on the efficacy of IDRF-based surgical decision and stepwise treatment intensification for patients with intermediate-risk neuroblastomas. We expect that this study will contribute to the establishment of a standard treatment for patients with intermediate-risk neuroblastoma.

**Trial registration:**

UMIN000004700, jRCTs051180203; Registered on December 9, 2010.

## Background

Neuroblastoma is the most common pediatric solid tumor, except for brain tumors [1], with about 200 newly diagnosed children each year in Japan. The prognosis of patients with neuroblastoma is strongly related to the age at diagnosis, stage, and biological factors [2–5]. In general, intermediate-risk tumors are mixed groups of localized unresectable tumors at diagnosis without *MYCN* gene amplification, and the survival of patients with distant metastases without *MYCN* gene amplification is less than 12 months. Because the intermediate risk group includes tumors with various clinical and biological characteristics, no clinical trial has been conducted in Japan, and a standard therapy has not been established. Therefore, a clinical trial was designed to establish a standard treatment for patients with intermediate-risk neuroblastoma.

It has been reported that complete resection rates decrease in localized cases with surgical risk factors and surgery-related complications increase [6]. The concept of image-defined risk factors (IDRF) has been proposed internationally as a criterion for estimating the risk of surgery from imaging findings and determining whether removal or biopsy is to be performed as initial surgery [7]. In this clinical trial, one of the goals is to reduce surgical complications while ensuring that the prognosis is not impaired. Therefore, the indication of initial surgery is determined based on IDRF. In addition, evaluations based on IDRF are conducted during delayed primary operations to reduce surgical complications as much as possible. Even if residual tumors are observed at the end of chemotherapy, treatment will be permitted to end if the risk of surgery was considered high according to the IDRF-based assessment. Chemotherapy will be performed for the intermediate risk group as a primary treatment after biopsy.

In the US CCG3881 trial, cisplatin (CDDP), doxorubicin (DXR), cyclophosphamide (CPA), and etoposide (VP-16) were used for patients with localized tumors in the intermediate risk group. According to the results of 228 patients with stage 3 cancer, the survival rate was 100% for favorable histology, and a 90% survival rate was reported for patients under 1 year of age with unfavorable histology. In contrast, the survival rate was 54%, and the prognosis was poor for patients over 1 year of age with unfavorable histologies [8]. Two treatment-related deaths were reported during remission.

In the SFOP report in France, chemotherapy with a combination of cyclophosphamide, vincristine, carboplatin, etoposide, and doxorubicin was performed for localized tumors [9]. A trial was conducted in 130 unresectable stage 3 cases, and the 3-year disease-free survival rate was reported to be 89%. Chemotherapy and treatment-related death after surgery have been reported as complications of one case each, and one case of acute renal failure after surgery and one case of Ewing’s sarcoma as a secondary cancer developed. Treatment with cyclophosphamide, thiotepa, etoposide, carboplatin, and deferoxamine was reported in the AIEOP of Italy [10]. The study was conducted in 48 unresectable stage 3 cancers in patients over 1 year of age, and the 5-year overall survival rate was 60%. However, this study included 10 patients with *MYCN* amplification, of whom seven experienced relapse and died. The treatment results for *MYCN* non-amplified cases were not described. Only one case of death was reported for each chemotherapy and surgery.

In the US CCG3881 study, patients with stage 4 cancer who were younger than 1 year of age, without *MYCN* amplification, showed good results, with a 3-year event-free survival (EFS) of 93% [11]. As complications, four treatment-related deaths were reported, and three patients died from infections. In France, 4–6 courses of the above treatment were performed for patients with stage 4 cancers who were younger than 1 year of age, and the 5-year EFS of 90% demonstrates that the treatment was well tolerated and successful in patients without bone metastasis [12]. In this study, among the patients without *MYCN* amplification and with bone metastasis, high-dose treatment was given to some who did not reach remission by initial treatment, and this report described two chemotherapy-related deaths. On the other hand, POG and CCG in the United States reported on stage 4 patients, at 12 to 18 months of age, respectively. The POG 9047 study treated patients with hyperdiploidy and without *MYCN* amplifications using CPA, DXR, CDDP, and VP-16 and reported a 4-year EFS of 92% [13]. One chemotherapy-related death was reported as a treatment complication. In addition, in the CCG 3891 study, treatment with CPA, DXR, CDDP, and VP-16 also resulted in an 86% 6-year EFS in all patients with stage 4 disease at 12–18 months of age. The CCG 3891 study assigned high-dose treatment and maintenance as an intensive treatment for patients who were 12–18 months of age without *MYCN* amplifications, but the efficacy of the high-dose treatment has not been shown, and there was no mention of complications in this report. The above findings demonstrate that, in other countries, the treatment for patients with intermediate-risk tumors consists of cyclic chemotherapy with regimens consisting of two to four agents, but the duration of treatment is inconsistent and the safety is insufficient.

Treatment outcomes for patients with intermediate-risk localized tumors in Japan have not been reported, and the treatment studies for localized tumors that have followed the low-risk infant neuroblastoma protocols 9405 and 9805 have shown only good results [14]. There are no reports on treatment results for infants who are 12–18 months of age with stage 4 tumors without *MYCN* amplification in Japan. For infants with stage 4 tumors under 12 months of age, 21 cases have been registered in the 9405 and 9805 studies, and the 4-year EFS was 88%.

To build upon the observations described above, this study aims to investigate an intensified regimen from the infantile neuroblastoma protocols 9405 and 9805 in Japan that used chemotherapy to treat patients with localized tumors. The chemotherapy regimen for patients with metastatic disease was the same as that used in Japanese infant neuroblastoma protocols 9405 and 9805. While VP-16 is frequently used in other countries, it was not used in this clinical trial due to the associated risk of secondary cancer. We have also found that high-dose chemotherapy with stem cell transplantation should not be given to patients at intermediate risk. However, the following changes are made in consideration of the characteristics of the target case. For the intensity regimen, we have decided to use LI-C consisting of VCR, CPA, and CBDCA for initial treatment for patients with stage 3 cancer, because cases of elder children and of patients of 12–18 months of age with unfavorable histology were also included. However, to prevent confusion, the initial treatment of patients with stage 3 tumors who were 1 year or older will only include the LI-B regimen consisting of VCR, CPA, and THP, which is used in infantile neuroblastoma protocols 9405 and 9805. Furthermore, some treatment responses were improved by using the LI-D regimen that consisted of increasing CPA, THP, and CDDP.

The duration of chemotherapy was 34 weeks in the COG 3881 trial and up to 32 weeks in the new 3961 trial [11, 15]. Furthermore, the duration of chemotherapies of the France SFOP trial and the Italy AIEOP trial ranged from 12 to 24 weeks, and the treatment periods were generally long [4, 10]. In this clinical trial, the chemotherapy period was shortened, and treatment was reduced by performing evaluations every three courses. On the other hand, if the treatment response was poor, we will use the LI-E regimen, which had an increased intensity. The treatment periods of this study will range from a minimum of three courses (9 weeks) to a maximum of nine courses (34 weeks).

For infants with stage 4 tumors without poor prognostic factors, we used the LI-D regimen of the Japanese infantile neuroblastoma protocol 9405/9805, to reduce treatment-related complications. During a total of five to six courses of treatment, distant metastasis is controlled, and surgery is performed when it is possible to remove the primary tumor. Although radiation therapy is not generally used as a treatment for patients at intermediate risk, cases of bone metastases in infants with stage 4 cancer have been reported by SFOP to have a poor prognosis [9]. We therefore plan to irradiate bone metastases for such patients; however, the indications for radiation therapy for the intermediate risk group remain controversial. Although radiation therapy will not be used in this clinical trial, patients with distant bone metastases are permitted to receive irradiation at the discretion of the institution, and this is not considered as protocol withdrawal.

## Methods/design

### Objectives

This trial aimed to achieve a good prognosis and reduce treatment complications by performing low-dose stepwise chemotherapy and evaluating operation timing, based on IDRF, for patients with intermediate-risk neuroblastoma. This study was conducted according to SPIRIT guidelines.

### Study design

**Fig. 1:**
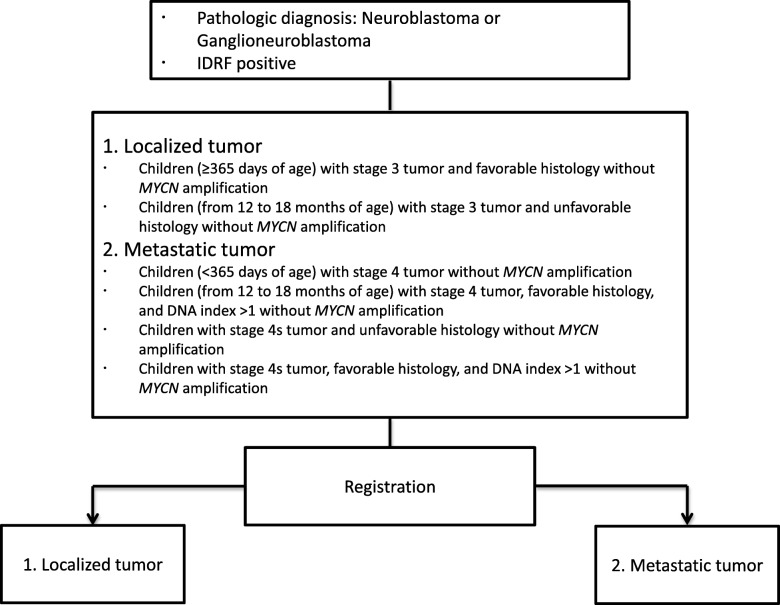
Study flow chart of JN-I-10

**Fig. 2:**
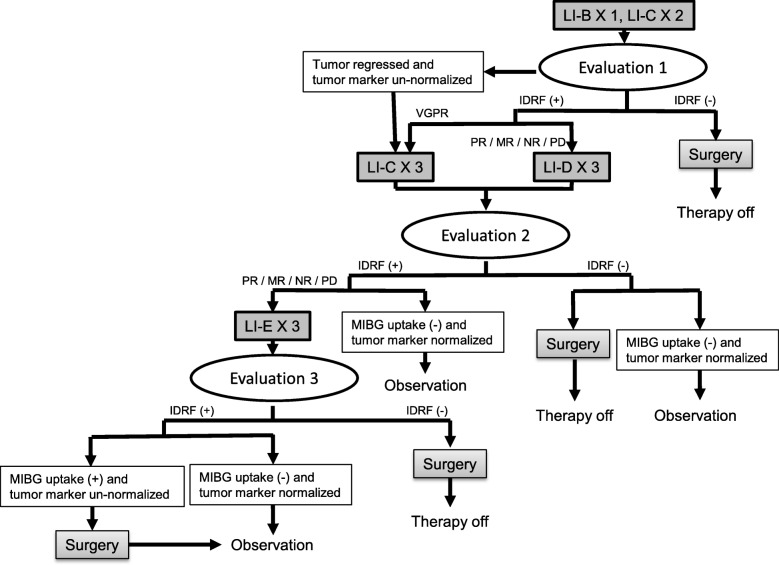
Study flow chart of localized tumors

**Fig. 3:**
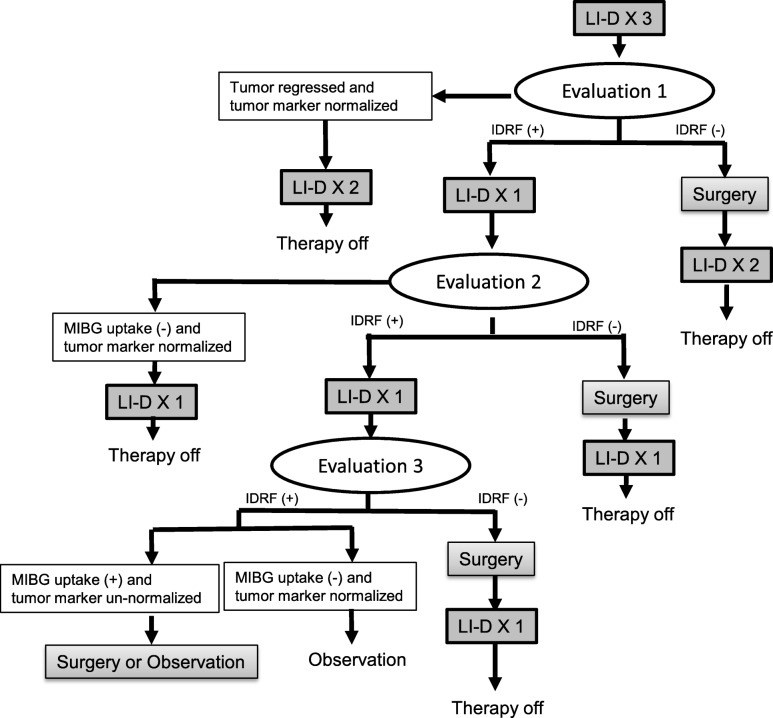
Study flow chart of metastatic tumors

The study is a prospective phase 2, single-arm study of the efficacy of IDRF-based surgical decision and stepwise treatment intensification for patients with intermediate-risk neuroblastomas. Figure 1 depicts the flow chart of the study. Patients of the localized tumor group will be evaluated by IDRF, and chemotherapy will be given to residual tumors after biopsy. Figure 2 every three courses of chemotherapy, the tumor will be evaluated by IDRF, and surgical removal will be performed when it is determined that there was no risk for surgery. The International Neuroblastoma Response Criteria will be used to determine chemotherapy efficacy. If up to nine courses show MIBG uptake or urinary vanillic mandelic acid (VMA) and homovanillic acid (HVA) do not reach normal values, tumor surgery will be limited to subtotal resection, and treatment will be considered complete. For the metastatic tumor group, a total of five courses of LI-D therapy will be performed and at each evaluation time point. Figure 3 primary tumors will be removed if IDRF is negative, regardless of the tumor marker. However, if it is possible to surgically remove the primary tumor after five therapy courses, a total of six courses of LI-D therapy will be given.

### Study setting

This trial has been approved by ethics review boards at 85 facilities.

Neuroblastoma is the very rare disease, and about 200 cases with neuroblastoma occur annually in Japan. It is said that only 10% of cases among them have an intermediate risk neuroblastoma, then the participants must recruit from many centers. The 85 participating facilities consist of 57 university hospitals, 22 Children’s hospitals and 6 public hospitals.

### Endpoints

Primary:

Three-year progression-free survival.

Secondary:

We will analyze the 3-year progression-free survival rates of the localized group and of the distant metastasis group. We will also analyze the 3-year overall survival of all eligible patients including those with localized disease, and those with distant metastases. Finally, we will calculate the incidence of adverse events.

### Participants

Inclusion criteria:

1. Age.
2. ≥ 0 days, < 18 years old.
3. Diagnosis.

- Pathological diagnosis with neuroblastoma or ganglioneuroblastoma.
- Increased urinary catecholamine (VMA, HVA) level and MIBG avid tumor, in cases without tumor biopsy.

(3) Stage, Prognostic factors.

- Stage 3, ≥ 12 months old, favorable histology, without *MYCN* amplification.
- Stage 3, 12–18 months old, unfavorable histology, without *MYCN* amplification
- Stage 4, < 12 months old, without *MYCN* amplification
- Stage 4, 12–18 months old, favorable histology, DNA index > 1, without *MYCN* amplification
- Stage 4S, unfavorable histology, without *MYCN* amplification
- Stage 4S, favorable histology, DNA index =1, without *MYCN* amplification

(4) Prior treatment.
  No history of chemotherapy or radiation, including treatment for other cancers.
(5) Organ failure.
  No serious organ damage that interferes with the treatment protocol.

① Performance status (PS).

Lansky PS ≥ 30.

② Hematopoietic function.

WBC ≥ 2000/mm [3].

③ Liver function.

ALT ≤300 IU/L and T-Bil ≤ 2.0 mg/dL.

Out of the limitations; neonatal jaundice and jaundice due to this disease; and increase in T-Bil due to constitutional jaundice.

④ Renal function.

< 5 years old: serum Cr ≤ 0.8 mg/dL.

5–10 years old: serum Cr ≤ 1.2 mg/dL.

10–18 years old: serum Cr ≤ 1.5 mg/dL.

⑤ Cardiac function.

There are no patients with heart disease who required treatment.

(6) Infection.

No active infections.

(7) Informed consent.

Provision of written informed consent.

### Exclusion criteria

1. Multiple cancers.
2. Patients who are pregnant, nursing, or possibly pregnant.
3. Patients with difficulty participating in the study due to mental illness or mental symptoms.
4. Severe complications or severe malformation.
5. Respiratory failure requiring respiratory management, such as intubation.
6. Disseminated Intravascular Coagulation (DIC)

### Dose and treatment regimens

The dose will be calculated based on body surface area as 1 m^2^ = 30 kg for patients whose body weight is less than 10 kg.

Regimen LI-B: 3-week interval.

Vincristine (VCR) 1.5 mg/m^2^/day day 1.

Cyclophosphamide (CPA) 600 mg/m^2^/day day 1.

Pirarubicin (THP) 30 mg/m^2^/day day 3.

Regimen LI-C: 3-week interval.

Vincristine (VCR) 1.5 mg/m^2^/day day 1.

Cyclophosphamide (CPA) 900 mg/m^2^/day day 1.

Carboplatin (CBDCA) 450 mg/m^2^/day day 1.

Regimen LI-D: 4-week interval.

Vincristine (VCR) 1.5 mg/m^2^/day day 1.

Cyclophosphamide (CPA) 900 mg/m^2^/day day 1.

Pirarubicin (THP) 30 mg/m^2^/day day 3.

Cisplatin (CDDP) 12 mg/m^2^/day days 1–5.

Regimen LI-E: 4-week interval.

Cyclophosphamide (CPA) 1200 mg/m^2^/day day 1.

Pirarubicin (THP) 40 mg/m^2^/day day 3.

Cisplatin (CDDP) 18 mg/m^2^/day days 1–5.

### Rationale for the number of enrolled subjects

The 5-year progression-free survival rate for intermediate-risk neuroblastoma in INRG is estimated to be 50 to 75% internationally [16]. On the other hand, for intermediate-risk neuroblastoma (stage 4, < 1 year old) with distant metastasis in Japan, the 3-year progression-free survival rate is 80 to 90%. Therefore, the expected 3-year progression-free survival rate of this study has been set to 85%. Since the international results refer to 5-year progression-free survival rate, the reference 3-year progression-free survival rate has been set to 75%. When the sample size is 65, a two-sided 95.3% confidence interval for a single proportion will extend 10% from the observed proportion for an expected proportion of 85%. The target sample size of this study is 73, including 10% patients that will be excluded from the analysis set. Logically, if the treatment outcome is equal to or better than this, this study will be considered to be as effective as a standard treatment.

### Statistical methods

An interim analysis is planned using alpha-spending function after 37 patients (the half of the target sample size) are evaluated. The progression-free survival rates and the confidence intervals are estimated using the Kaplan-Meier method. For adverse event, the worst grade over the entire course of each adverse event in each subject is summarized.

### Study population

All subjects excluding patients with serious violations will be included in this study.

Patients who received anticancer drugs other than those included in the protocol treatment regimen and who received folk remedies for antitumor effects were judged as serious violations.

### Regular monitoring

Regular monitoring is conducted twice a year in order to confirm whether the test is performed safely as planned and in accordance with the protocol, or whether data is collected appropriately. The results of the interim analysis are submitted as an interim analysis report from the data center to the Efficacy and Safety Evaluation Committee, where they are examined for the continuation of the test and the availability of the results.

### Trial status

This study opened recruitment in December 2011, with a planned last follow-up in December 2023. As of December 2020, 73 subjects will be enrolled.

## Discussion

Intermediate-risk neuroblastoma is a rare disease, and few clinical trials have been reported. In addition, since intermediate-risk tumors are heterogeneous and show various tumor dynamics, treatment selection is often difficult. On the other hand, recent reports from the COG group show that the 3-year overall survival of patients with intermediate-risk tumors is as good as 96%, which suggests the possibility for further chemotherapy reduction [15]. We will not use etoposide for chemotherapy or high-dose treatment with stem cell rescue, and we plan to reduce the risk of secondary cancer and complications. With regard to drug doses, patients with localized tumors will receive initial combination therapy with up to three drugs, and we aim to reduce side effects by gradually increasing the intensity. In the induction therapy for high-risk cases in Japan, the 05A3 protocol consisting of CPA, CDDP, VCR, and THP in combination is used, and a high remission rate has been achieved [17]. For metastatic cases, to reduce side effects, we will reduce the CPA and CDDP more than this 05A3 and calculate the dose by weight for infants younger than 1 year of age. We aimed to achieve both good outcomes and reduced side effects.

The tumors of patients in the intermediate risk group include IDRF-positive tumors with surgical risks. Surgery will be delayed as a primary operation after chemotherapy, but few reports from previous clinical studies have described tumor removability and surgical complications in intermediate-risk cases. The operation time decision verifies whether the operation complication can be reduced based on the objective IDRF rather than the subjectivity of the surgeon. The trial design is a non-randomized phase 2 trial, because it is difficult to increase the number of patients with this rare disease.

The intermediate-risk patients, who were assigned risk classification accordingly, consisted of two subgroups with different characteristics: a localized group and a distant metastasis group. Unfortunately, the number of patients expected in Japan is not large. From previous reports, the number of newly diagnosed patients in Japan has been reported to be 10 patients/year for the localized group and 5.5 patients/year for the metastatic group. Therefore, with approximately 15 patients in both cases, it is difficult to conduct clinical trials. Therefore, in this study, we will explanatively estimate the results of the treatment group for intermediate-risk patients in which the limited and remote groups were combined, and we will select the number of cases based on accuracy. Hence, the number of cases is set such that the 95.3% confidence interval for the expected 3-year progression-free survival rate of 85% is ±10%. In this case, the lower limit of the 3-year progression-free survival rate is 75%, which can be compared with the threshold 3-year progression-free survival rate of 75%. Lawless’s size design formula requires 65 patients or more and adds 10% ineligible cases; as such, 73 patients are required. Assuming that the annual expected number of cases is 15, 4.9 years will be required to enroll 15 patients; therefore, the registration period was originally planned it would be 5 years. The actual registration period may increase or decrease depending on the number of actual ineligible cases. In fact, we found that patients with moderate-risk neuroblastoma were very rare and that recruiting participants could take years, so we revised the registration period to 10 years in 2014.

The results of our clinical trial will allow the establishment of an efficacious and safe standard treatment for patients with intermediate-risk neuroblastoma in Japan.

This study is the first clinical trial of the efficacy of IDRF-based surgical decision and stepwise treatment intensification for patients with intermediate-risk neuroblastomas. We hope that this study will contribute to the establishment of standard treatment for patients with intermediate-risk neuroblastoma.

## Data Availability

Data sharing does not apply to this article as this study is still open for patient enrollment. However the datasets are available from the corresponding author on reasonable request.

## Abbreviations

CDDP: Cisplatin
CPA: Cyclophosphamide
DXR: Doxorubicin
EFS: Event-free survival
HVA: Homovanillic acid
IDRF: Image-defined risk factors
JCCG: Japan Children’s Cancer Group
PS: Performance status
VMA: Vanillic mandelic acid
